# Abnormal origin of internal thoracic artery from the thyrocervical trunk: surgical considerations

**DOI:** 10.1186/1749-8090-7-63

**Published:** 2012-06-29

**Authors:** George Paraskevas, Konstantinos Natsis, Maria Tzika, Orestis Ioannidis, Panagiotis Kitsoulis

**Affiliations:** 1 Department of Anatomy, Medical School, Aristotle University of Thessaloniki, Thessaloniki, Greece; 2 Department of Anatomy-Histology-Embryology, Medical School, University of Ioannina, Ioannina, Greece

**Keywords:** Internal thoracic artery, Internal mammary artery, Thyrocervical trunk, Anomalous origin, Graft

## Abstract

An unusual case of left internal thoracic artery (ITA) origin from the thyrocervical trunk (TCT) was detected during routine cadaver dissection. The variability of origin and course of ITA has less or more frequently been documented in the literature. However, the ITA origin from the TCT on the left side has been detected less commonly, making its dissection and preparation during coronary artery bypass grafting surgery more difficult. We discuss the ITA origin and course variability as well as clinical significance of the present variant, reviewing the relative literature. The objective of our study is to exhibit a rare ITA origin in order to provide a more accurate knowledge of such variations.

## Background

Internal thoracic artery (ITA) is a trait of important anatomical variability and clinical significance. It is widely utilized for coronary artery bypass grafting (CABG) surgery. The ITA’s anatomic characteristics, such as distinct intrathoracic course and anatomical vicinity with the heart [1], its long-term patency [1-4], the long-term survival rate [2,4] and post-operative quality of life [5] render it as an excellent arterial graft for myocardial revascularization.

Anomalous origin of the ITA has continually been reported in the literature. Unilateral [4,6,7] or bilateral [8] ITA origin from the lateral part of the subclavian artery (SCA) has been mentioned in less than 1 % of the studied cases [6], while the possible common origin of the ITA and branches of the thyrocervical trunk (TCT) is clinically underestimated in general. Does the ITA common origin from the TCT affect the implant’s suitability and survival? The variability in ITA origin gives birth to intense interest and extensive research in order to avoid surgical complications and clinical discomfort. We study the anatomic relationship of common ITA and TCT origin, as well as its possible consequences intra- and post-operatively.

## Case report

**Figure 1:**
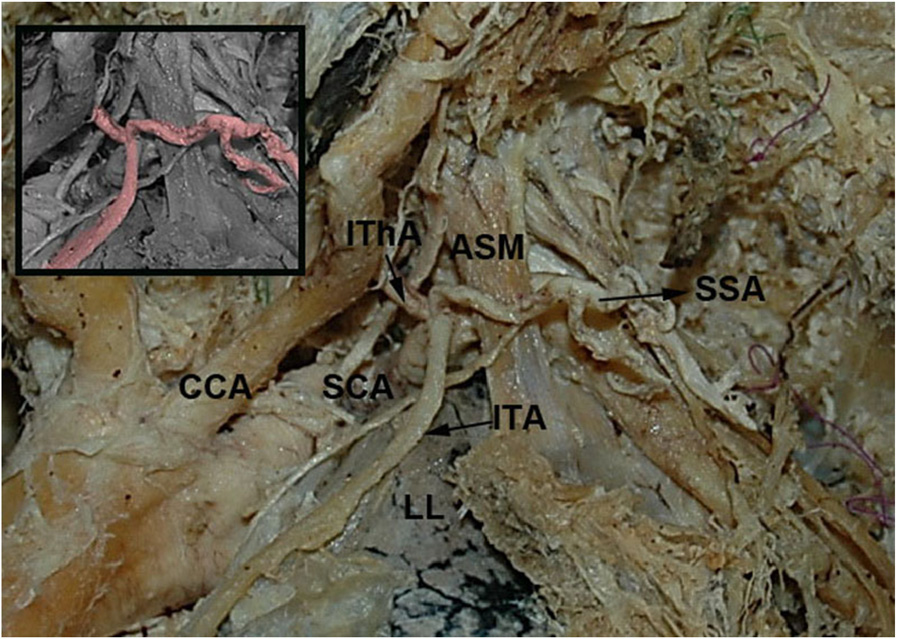
**Common origin of the left internal thoracic artery (ITA) and thyrocervical trunk (TCT).** The ITA arises from the subclavian artery (SCA) as a common trunk with the inferior thyroid (IThA) and suprascapular (SSA) arteries. CCA: common carotid artery, ASM: anterior scalene muscle, L: left lung.

An unusual case of common origin of ITA and TCT was encountered during routine gross anatomy dissection undertaken for education and research purposes in a formalin-fixed 79-year-old female cadaver. By means of typical methods of anatomic dissection and preparation, we dissected carefully the main branches of the aortic arch with their ramifications. Both SCAs were dissected preserving their branches and neighboring structures, while the ITA throughout its course was bilaterally exposed. Whereas on the right side ITA exhibited no variations, on the left side we came across an unusual origin of ITA from the TCT, the latter one being composed of a common trunk for the inferior thyroid and suprascapular artery, with the ascending cervical artery arising from the inferior thyroid artery. Left ITA after originating from TCT directed arcuately downwards passing anterior to SCA following afterwards its usual course and providing its normal branching pattern (Figure 1). The cause of specimen’s death was unrelated to the present study. The documented vascular variation was not accompanied by other abnormalities of the neck and thorax regions. Furthermore, no evidence of previous surgical procedures, traumatic injuries or pathologic processes was present. The precise morphology and topography of the detected variational anatomy was documented by photographs.

## Discussion

Internal thoracic or mammary artery is the commonly used term for the vessel description, although sternal or parasternal [9] and Vineberg artery [10] constitute alternative denomination proposed in the literature. The artery was initially called “internal mammary artery” by Basle Nomina Anatomica in 1895 [11], whilst later as official term was utilized the “internal thoracic artery” by Paris Nomina Anatomica in 1955 [12]. ITA arises from the anteroinferior aspect of the SCA at about the similar level as the TCT. The artery passes inferomedially into the thorax with the cervical part providing no branches. It enters the thorax behind the cartilage of the first rib and usually behind the sixth cartilage it divides into its two terminal branches, the musculophrenic and the superior epigastric arteries. Inside the thorax, ITA provides mediastinal, sternal, pericardial, perforating and anterior intercostals branches [13-15]. Although the termination level ranges between the third and seventh rib [3], ITA commonly terminates at the level of the sixth intercostal space, dividing into its terminal branches [1,3,16]. Henriquez-Pino et al. [16] encountered the presence of a third terminal, diaphragmatic ITA branch in 7 % of the cases, whereas trifurcation of the artery was also found in 12.5 % [17]- 61.3 % [18], including a xiphoid branch. There has also been reported a stepladder anastomosis pattern of a bifurcated right ITA [19].

Complicating the arterial anatomy, a lateral ITA branch called “lateral costal branch” or “accessory ITA” may also originate from the existing ITA [20,21] and after an oblique course in the inner surface of the anterolateral thoracic wall anastomoses with the neighboring intercostal arteries. That accessory ITA may originate in an incidence of 4.54 % [21], 10.8 % [20] or 15 % [16]. The ITA’s parietal branches, thus the anterior intercostal, sternal, perforating and mediastinal arteries may arise separately or from a common trunk and the branching patterns have been classified into four types for the case of common trunk and four types for the separate branches [2]. The average artery length varies from 18.07 cm [18] to 20.4 cm [16]. A longer vessel course is detected in left sides and in male population [3,16] presumably due to differences in thoracic dimensions. ITA lies in close proximity to the phrenic nerve risking partial iatrogenic paralysis of the diaphragm during ITA harvesting for CABG [1].

Nizanowski et al. [22] mentioned that in 11.4 % of the cases studied the ITA was found abnormal or absent, while cases of hypoplastic arteries have also been reported in the literature [23]. Anomalous unilateral [4,6] or bilateral [8] ITA origin from the third portion of the SCA has also been encountered in the literature in less than 1 % of the cases, thus 0.78 % [24], 0.5 % [25], 0.83 % [6], increasing the vessel injury risk during percutaneous subsclavian catheterization [8]. The graft patency may also be reduced in case the ITA used for CABG surgery arises from the third SCA portion due to vessel traction [4].

It has been documented that in more than 79 % of the cases the ITA arises from the first part of the SCA [1,16,24]. It has been particularly indicated that in 2.6 % [24] or 16 % [16] the left ITA originated in common with the suprascapular artery, in 5 % in common with the suprascapular and transverse cervical arteries [16], in 0.5 % with the transverse cervical alone [24], also in 0.5 % with the inferior thyroid artery [24], in 4 % with the ascending cervical and inferior thyroid arteries [16], whereas only in 0.1 % the artery has a common origin with the transverse cervical and suprascapular arteries [24]. Furthermore, the left ITA had a common origin with the suprascapular and inferior thyroid arteries in 2 % [16], with the ascending cervical and suprascapular arteries in 1 % [16], in another 1 % left ITA co-originated with the ascending cervical artery [16] and in 1 % [16] or 7.4 % [24] the left ITA originated in common with the four TCT branches. Daseler et al. have noticed that the left ITA originated from the second portion of SCA, the axillary artery and the superior intercostal artery in 2.9 %, 0.3 % and 0.3 %, respectively [24]. Moreover, Henriquez-Pino et al. found that the right ITA originated as a single trunk from the SCA in 95 % of total cases [16]. Lischka et al. (1982) [26] mentioned that in a considerable incidence of 10 % the left ITA originated from the TCT, while the right ITA had the same origin only in 2 %. They also highlighted that in some cases ITA may give off branches usually classified as TCT branches. Puri et al. [1] reported that the right ITA arose in common with the TCT in 4 % and Wisniewski et al. [3] in 6.2. Uemura et al. [27] additionally noticed that the ITA originated from the TCT in 11.8 % of the 110 cases in which the SCA passed posteriorly to the anterior scalene muscle, as normal.

The ITA is found to be an artery with elastic properties [28,29] that is resistant to atherosclerosis [30,31], although cases of diseased arteries have been reported in the literature [32,33]. This anatomically medium-sized artery receives sympathetic fibers in its adventitia [28] and has routinely been harvested as a suitable conduit for the myocardium ischaemia relief, showing an increased patency compared to the saphenous vein grafts [34]. During CABG surgery it is important to reassure the conduit length, course, and mobility within the surrounding structures in order to prevent traction and angulation [4].

The curved direction of the ITA-TCT common trunk makes the implant particularly vulnerable to traction and kinking, risking blood flow blockage and congestion. The usual CABG technique includes the free implant placing into an artificial tunnel in the myocardium [35]. Presumably, our curved ITA cannot be easily placed into the artificial tunnel, causing technical problems to the surgeon. The skeletonization technique, that has arisen debate in the literature [36,37], could be proven suitable in our case as it involves the separation of the artery from any surrounding tissue [37], providing a longer conduit [38,39] and possibly avoiding the intra-operative traction. Both pedicled and skeletonized left ITAs provide optimal patency [40], while the advantages of this new technique include decreased postoperative pain and dysesthesia [37].

**Figure 2:**
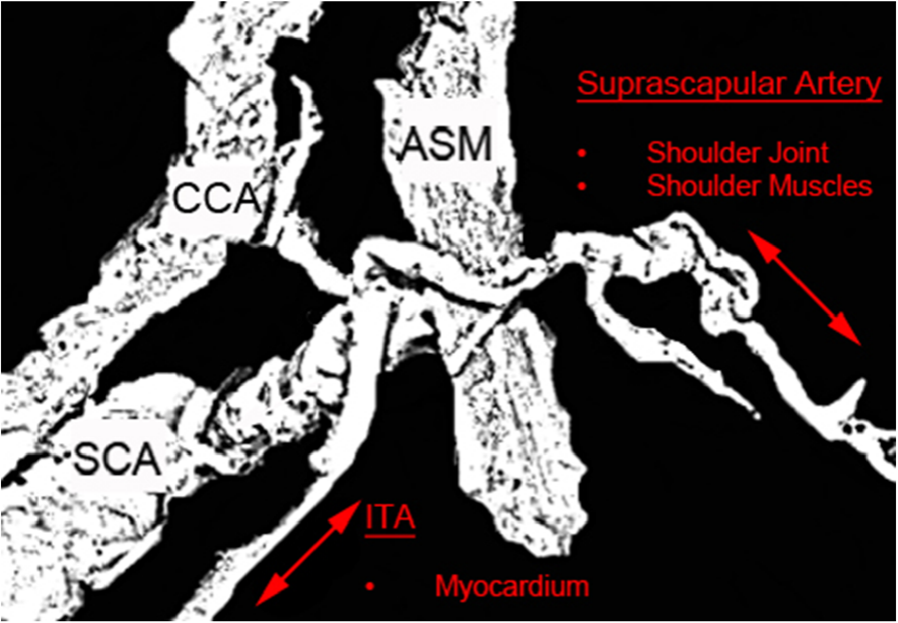
**Schematic representation of the blood steal phenomenon.** Both myocardium and shoulder area may present blood supply deficiency, as the internal thoracic artery (ITA) and the suprascapular artery arise from a common trunk and receive a limited amount of blood. CCA: Common Carotid Artery, SCA: Subclavian Artery, ASM: Anterior Scalene Muscle.

Anatomic factors, such as the co-origin of other arteries together with the ITA that is utilized as a CABG graft, could be responsible for blood flow diversion and may lead to symptomatic coronary steal phenomenon (Figure 2). The syndrome has been mentioned to originate from existent mammary side branches [41], proximal SCA stenosis [42] and ITA-TCT common trunk [43]. As a result, post-operative pain or even the need for re-operation may arise, as the implant may not be able to meet the myocardium oxygen demands [43]. The phenomenon co-exists with an unexpected blood flow inversion from the muscular regions that are normally supplied by the TCT branches. The number of the TCT branches originating in common with the ITA, may influence the patient’s symptomatology. An insufficient blood flow could ultimately lead to muscle palsy and joint movement limitations. It has been mentioned that in a patient underwent CABG intervention, the common origin of the ITA, inferior thyroid and suprascapular arteries, as it occurs in our case, is capable of causing pain and significant discomfort during shoulder movements. Manual exercise testing and angiographic results may prove the diagnosis postoperatively and vessels’ ligation could provide solution [43].

The anatomy, patency and ramification pattern of ITA can be visualized during routine pre-operative angiography, although its safety and necessity is debatable [4]. The risk of the procedure is not neglectable and some authors insist on its avoidance [43]. Based on our review of the literature and the frequency of the anatomical variation studied, we suggest preoperative selective angiographic evaluation involving the SCA-ITA junction, except from the cases in which serious contraindications are present. In cases where such an evaluation indicates the presence of common ITA-TCT trunk, another implant option or surgical approach could be preferred.

## Conclusions

It is obvious that the presence of a common ITA-TCT trunk may complicate the surgical grafting procedure. The ITA is widely utilized as a great conduit for myocardial revascularization and its origin variability is of great surgical significance. The common origin of the left ITA and TCT branches could be undetected intra-operatively, although angiography could appear helpful. The awareness of such ITA’s anatomical variations, potentially causing surgical complications, is essential for thoracic and cardiovascular surgeons in order to avoid post-operative discomfort.

## Abbreviations

ITA, Internal thoracic artery; TCT, Thyrocervical trunk; SCA, Subclavian artery; CABG, Coronary artery bypass grafting.

## Competing interests

The authors declare that they have no competing interests.

## Authors’ contributions

GKP, KN, and MT carried out the study design, data analysis and writing, GK and MT provided the schematic drawing. GKP and KN had performed data collection and cadaver dissection. OI and PK made a critical review of the manuscript. All authors have read and approved the final manuscript.
